# Case report: an area postrema syndrome revealing a neuromyelitis optica spectrum disorder associated with central nervous system tuberculosis in a young Togolese (black African) woman

**DOI:** 10.1186/s12883-019-1287-5

**Published:** 2019-04-10

**Authors:** Kossivi Apetse, Josué Euberma Diatewa, Jean Joel Dongmo Tajeuna, Yaovi Mawuéna Dansou, Rolph Bakoudissa, Kokouvi Panabalo Waklatsi, Damelan Kombate, Komi Assogba, Agnon A. Koffi Balogou

**Affiliations:** 1 Faculte des Sciences de la Sante, Universite de Lome, BP 1515, Lome, Togo; 2 Service de Neurologie, CHU CAMPUS de Lome, 03 BP 30284, Lome, Togo; 3 Service d’imagerie médicale, CHU Sylvanus Olympio, BP 18, Lome, Togo

**Keywords:** Area postrema syndrome, Neuromyelitis optica spectrum disorders, Tuberculosis, Togo, Africa

## Abstract

**Background:**

Area postrema syndrome (APS) is considered to be one of the most specific clinical presentations of neuromyelitis optica spectrum disorders (NMOSDs). In sub-Saharan Africa, NMOSDs and even more so those revealed by an APS, are rarely reported. However, studies among mixed populations have shown that NMOSDs disproportionately affect black people with relatively more frequent encephalic involvement. We report a case of APS revealing an NMOSD associated with central nervous system (CNS) tuberculosis in a young Togolese woman residing in Togo (West Africa).

**Case presentation:**

A 28-year-old Togolese woman was admitted for left hemibody sensory problems with ataxia. These problems were observed while the patient was hospitalized for a few days in the hepato-gastroenterology department for persistent vomiting, abdominal pain and hiccups lasting for about a month. The examination confirmed left hemibody ataxia with nystagmus when looking to the left, pronounced left osteotendinous reflexes, and left hemibody hypoesthesia up to the base of the neck. Encephalic magnetic resonance imaging (MRI) showed a hypersignal lesion in the bulbar more lateralized on the left in the fluid-attenuated inversion recovery sequence, not enhanced after a gadolinium injection. Biological assessment showed the presence of *Mycobacterium tuberculosis* deoxyribonucleic acid in the cerebrospinal fluid and a sedimentation rate of 120 mm in the 1st hour. The result of the anti-AQP4 antibody test was positive. Two months from the onset of digestive problems with Lhermitte’s sign and hand and foot contracture access without vesico-sphincter problems were established. Cervical medullary MRI showed an additional intramedullary hypersignal lesion in the T2 sequence at the C2 level, not enhanced after a gadolinium injection. A second course of intravenous corticosteroids was administered, and anti-tuberculosis treatment was continued. The outcome was favorable. After 8 months of anti-tuberculosis treatment, the patient started immunosuppressive therapy (azathioprine 50 mg twice daily) to limit the risk of recurrence of NMOSD.

**Conclusion:**

The recognition of an APS is an additional challenge for the diagnosis of NMOSDs, especially in countries with limited resources. CNS tuberculosis must be tested when faced with an NMOSD because it seems to be a major cause.

## Background

Neuromyelitis optica (NMO), an autoimmune, inflammatory and demyelinating disease of the central nervous system (CNS) that is distinct from multiple sclerosis, has long been considered a monophasic optic medullary disease [1, 2]. However, with the discovery of anti-aquaporin-4 (anti-AQP4) autoantibodies that are highly specific to the disorder, the concept of NMO spectrum disorders (NMOSDs) has emerged. This concept includes other clinical presentations, including encephalic symptoms and clinical forms evolving into outbreaks. The area postrema syndrome (APS) consists of hiccups, nausea, and/or uncontrollable vomiting for several days in connection with an area postrema attack, a bulbar region, and an emetic reflex center. This region is particularly rich in aquaporin-4, the target of anti-AQP4 responsible for NMOSDs. As a result, this syndrome is considered to be one of the most specific clinical presentations of NMOSDs [3–5]. The diagnosis of this neurological syndrome is not always easy, and digestive symptoms are the most prominent. In sub-Saharan Africa, NMOSDs and even more so those revealed by an APS, are rarely reported. However, studies among mixed populations have shown that NMOSDs disproportionately affect black people with relatively more frequent encephalic involvement [6]. We report a case of APS revealing an NMOSD associated with CNS tuberculosis in a young Togolese woman residing in Togo (West Africa).

## Case presentation

A 28-year-old Togolese woman of Ewe ethnic origin working part-time in a prison setting with no previous history of disease was admitted in August 2017 for left hemibody sensory problems with ataxia. These problems were observed while the patient was hospitalized for a few days in the hepato-gastroenterology (HGE) department. She had been referred to the HGE department for vomiting, abdominal pain and persistent hiccups lasting for about a month, which were thought to be due to gastritis with multiple ulcers based on a digestive endoscopy. Prior to admission to the HGE department, she initially received anti-ulcer and antiemetic drugs, but the outcome was marked by persistent vomiting and the appearance of episodes of prolonged loss of consciousness. Before the appearance of digestive problems, the patient presented with headaches and auditory and visual hallucinations due work-related stress. The examination also confirmed the existence of evening fever, weight loss without cough and secondary amenorrhea unrelated to pregnancy.

**Fig. 1:**
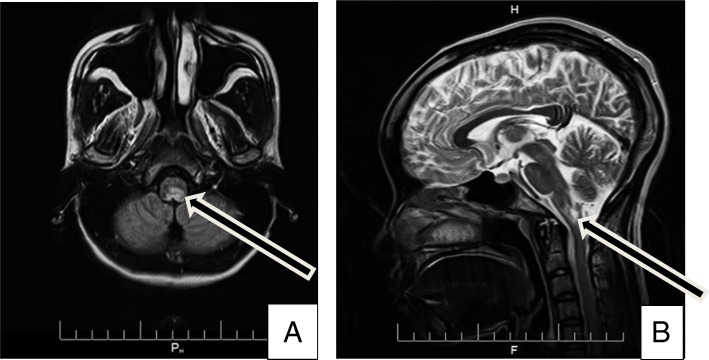
Axial Fluid Attenuated Inversion Recovery (**a**) and sagittal T2-weighted magnetic resonance imaging sequences demonstrate a bulbar left more lateralized lesion (arrows)

**Fig. 2:**
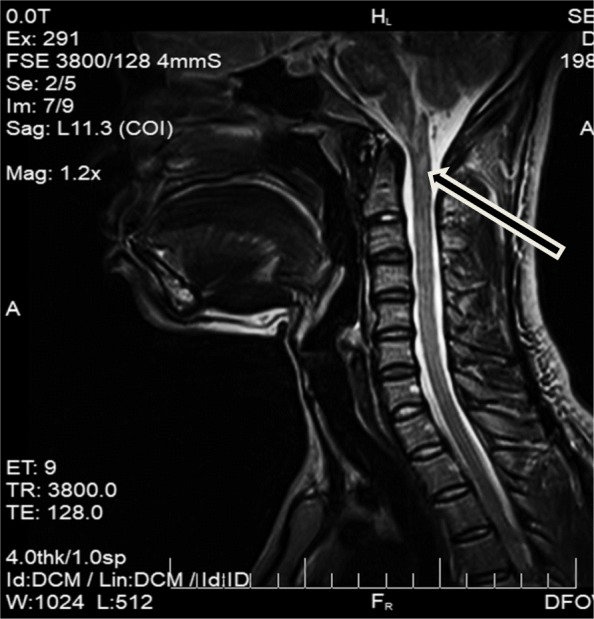
Cervical medullary magnetic resonance imaging showing an additional intramedullary hypersignal lesion at C2 level (arrow) in T2 sequence 3 months later

On day 1 of neurological problems, an examination confirmed the persistence of digestive symptoms, apyrexia, the existence of a headache, left hemibody ataxia with nystagmus when looking to the left, pronounced left osteotendinous reflexes, and left hemibody hypoesthesia up to the base of the neck. Encephalic magnetic resonance imaging (MRI) showed a hypersignal lesion in the bulbar more lateralized on the left in the fluid-attenuated inversion recovery (FLAIR) sequence not enhanced after a gadolinium injection (Fig. 1). The infectious assessment showed a normal pulmonary X-ray, a negative human immunodeficiency virus (HIV) serology, negative plasmodium tests but the presence of *Mycobacterium tuberculosis* deoxyribonucleic acid (DNA) in the cerebrospinal fluid (CSF) (using GeneXpert) with normal cytochemistry and a sedimentation rate (SR) of 120 mm in the 1st hour. The pregnancy test was negative. On day 3 of the neurological problems, digestive problems subsided, and even though the results of the examinations were not yet compiled, the patient presented three episodes of cardiopulmonary arrest with a fever of 39 °C without an infectious contact point. She successfully benefitted from orotracheal intubation with broad-spectrum antibiotic treatment and intravenous corticosteroids in the intensive care unit. In the presence of the digestive symptomatology with a bulbar lesion, we alluded to an APS within the framework of an NMOSD, and a sample for the anti-AQP4 antibody tests was sent to Paris, France. Upon leaving the intensive care unit after 72 h, anti-tuberculosis treatment was established. The outcome was favorable with apyrexia, a modification in the nystagmus and an improvement in the ataxia. Subsequently, the result of the anti-AQP4 antibody test was positive (indirect immunofluorescence on transfected cells, anti-AQP4 Euroimmun reagent, CERBA file No. 17 T0483884 of 01/09/2017). In September, 2 months from the onset of digestive problems, with Lhermitte’s sign and hand and foot contracture access without vesico-sphincter problems were established. At the neurological examination, the osteotendinous reflexes were pronounced. Cervical medullary MRI showed an additional intramedullary hypersignal lesion in the T2 sequence at the C2 level without contrast enhancement after a gadolinium injection (Fig. 2). In addition to the symptomatic treatment of spasticity, a second course of intravenous corticosteroids was administered, and anti-tuberculosis treatment was continued. The outcome was favorable, and the patient resumed her usual activities as of February 2018, which was 8 months after the first onset. After 8 months of anti-tuberculosis treatment, the patient was started immunosuppressive therapy (azathioprine 50 mg twice daily) to limit the risk of recurrence of NMOSD.

## Discussion and conclusions

We report a case of NMOSD in a black African patient who has always lived in her country Togo. To the best of our knowledge, this is the first case to report CNS tuberculosis with positive AQP4-IgG NMOSD even from regions with a high incidence of tuberculosis, such as South Africa. The diagnosis of NMOSD was suspected from the presence of radioclinical features of APS, a syndrome reported in 7–44% of patients suffering from an NMOSD and considered highly suggestive of NMOSD. The area postrema, the emetic reflex center, is located outside the blood-brain barrier, making it more accessible to AQP4-IgG attacks. However, the digestive symptoms at the forefront of this syndrome often delay the diagnosis of NMOSD as digestive pathologies are alluded to initially. In addition, the lack of knowledge of these conditions and the limits of the technical capacity make this diagnosis more difficult in our context, as evidenced by our patient’s course of therapy. In a practical way, an APS should be alluded to and a bulbo-cervical MRI should performed in view of persistent vomiting and/or hiccups without an obvious digestive cause. The prolonged loss of consciousness with cardiopulmonary arrest was linked to bulbar center dysfunction. As a result, the diagnosis of an APS is considered urgent to take the appropriate therapeutic measures to avoid cardio-respiratory arrest.

The detection of a strain of *Mycobacterium tuberculosis* in the patient’s CSF using GeneXpert (sensibility of approximately 27%, specificity of approximately 99% [7]) alluded to the existence of an associated CNS tuberculosis. This hypothesis was more plausible since there were symptoms (weight loss, evening fever, amenorrhea, SR of 120 mm in the first hour), although nonspecific, that were within the framework of a tuberculin permeation. The positive outcome when using anti-tuberculosis treatment also reinforced this hypothesis. In a comparative study, Feng et al. reported that anti-tuberculosis treatment in China provided better neurological recovery in patients with corticosteroid-refractory NMOSD (the diagnosis criteria of these NMOSD cases did not include AQP4-IgG data). They concluded that CNS tuberculosis was a major cause of NMOSD, suggesting that some NMO cases are caused by a direct CNS infection with tuberculosis [8]. Thus, tuberculosis should be actively tested for when faced with an NMOSD. However, another possible explanation for tuberculosis-related NMO relates to a nonspecific adjuvant effect of tuberculosis that amplifies the immune response. Immune dysregulation from NMOSDs may result from the interaction of a predisposed host with environmental triggers, such as infections by *Mycobacterium tuberculosis*, HIV, and Epstein Barr virus [9, 10]. It is also known that other autoimmune diseases may present in association with NMOSDs, including systemic lupus erythematosus and Sjögren’s syndrome [11]. Jarius et al reported that AQP4-IgG was detectable in 31/40 (78%) patients with connective tissue disorders and NMOSDs [12]. The mechanism by which these autoimmune processes occur together remains unclear, but it is possible that they are phenotypes of a genetic background susceptible to developing humoral autoimmunity [13].

The recognition of an APS is an additional challenge for the diagnosis of NMOSDs, especially in countries with limited resources. Tuberculosis of the CNS must be tested when faced with an NMOSD because it seems to be a major cause. In sub-Saharan Africa, the high frequency of tuberculosis contrasts with the few cases of NMOSD that are reported.

## Abbreviations

anti-AQP 4: anti-aquaporin 4
APS: Area postrema syndrome (APS)
CNS: Central nervous system
DNA: Deoxyribonucleic acid
FLAIR: Fluid attenuated Inversion Recovery
HGE: Hepato-gastroenterology
MRI: Magnetic resonance imaging
NMO: Neuromyelitis optica
NMOSD: NMO spectrum disorder
SR: Sedimentation rate
